# The Impact of Non-caloric Sweeteners on Male Fertility: A Systematic Review and Narrative Synthesis in Rodent Models

**DOI:** 10.3389/fnut.2022.854074

**Published:** 2022-06-28

**Authors:** Michelle L. Kearns, Fionn MacAindriu, Clare M. Reynolds

**Affiliations:** ^1^School of Public Health, Physiotherapy and Sports Science, University College Dublin, Dublin, Ireland; ^2^Conway Institute/Institute of Food and Health, University College Dublin, Dublin, Ireland

**Keywords:** artificial sweetener, male fertility, sperm quality, systematic review, rodent model

## Abstract

Understanding the factors which influence fertility is essential for developing appropriate nutritional recommendations for couples trying to conceive. Non-caloric sweeteners (NCS) are increasing in the food chain and despite being no/low calorie, several adverse metabolic consequences have been attributed to their consumption. Their effects on reproduction have been relatively under-researched, particularly in males. This review aims to systematically review the literature for evidence of the effect of NCS on male fertility in rodents, with sperm parameters (sperm quantity and quality) assessed as primary outcomes. Given the lack of information available in humans this review has been carried out using evidence from rodent models. Risk of bias assessment was carried out using the Syrcle risk of bias tool. Nine studies met the inclusion criteria. Forty-four percent showed a negative effect of NCS on male reproductive parameters compared with controls. The effects of NCS on fertility have been conflicting and selected studies have been heterogeneous in relation to study design. It is unclear if NCS has an impact on male reproductive function. There is a need for randomized controlled trials using a standardized protocol for analysis, to formulate a clear message in terms of male fertility.

## Introduction

Infertility is a growing public health concern worldwide, affecting ~168 million individuals and 48 million couples worldwide (1), with male infertility accounting for up to 50% of cases (2). Male infertility is associated with poorer health outcomes such as increased risk of cancer and a lower life expectancy, in addition to poorer psychological and marital stress. It is therefore imperative that evaluation of male fertility in addition to female fertility should be conducted, however it is currently not performed in at least 18% of cases, compromising the couple's fertility prognosis and potentially missing the opportunity to improve health outcomes (3).

There are numerous driving factors of male infertility including; hypogonadism, testicular cancer, injury/trauma, lifestyle related factors and diseases, infection, radiation, obstruction and idiopathic male infertility, which can be caused by environmental factors, reactive oxygen species and genetic abnormalities (4). Drivers of male infertility, such as lifestyle factors may be modifiable and may reduce the prevalence of male infertility (5). Numerous studies have investigated the link between the western diet and male infertility, with links observed between increased saturated fat intake (6), sugar-sweetened beverages (SSB) (7) dairy intake (8), and male infertility.

The Western diet, high in fat, salt and sugar is well known for its role in driving obesity rates and metabolic diseases (9). Many strategies have been implemented to tackle rising obesity rates, including the use of non-nutritive sweeteners to substitute sugar intake, thus reducing overall calorie intake. Consumption of NCS has increased in recent years and are popular among consumers as a sugar substitute, providing intense sweetness with minimal or no energy (10). NCS are abundant in our current food supply and are widely found in diet carbonated beverages, yogurts, dairy, chewing gum and even toothpaste, with the number of products containing these NCS continuously growing (11–13). Currently, the approved sweeteners with evidence for safe consumption and assigned acceptable daily intake (ADI), in the European Union (EU) are; acesulfame-K, aspartame, cyclamates, saccharin, sucralose, neohesperidine DC (NHDC), neotame, salt of aspartame-acesulfame and advantame (used only in bakery products for special nutritional uses).

As consumption of NCS continues to rise, the use of NCS remains controversial, with studies in rodents showing adverse effects, including increased oxidative stress (14, 15), changes in behavioral parameters (16) and elevated glucose concentrations (17). In addition, the effect of NCS on reproduction has been relatively under-researched, particularly in males. However, several studies in both human and animal studies have demonstrated negative implications of artificial sweetener use during pregnancy, both on maternal and offspring health. Cho et al. (18) showed that obese rats who consumed stevia, had a lower fertility index than obese control and obese-aspartame groups, suggesting that certain sweeteners in addition to obesity may impact the ability of rats to conceive. Plows et al. (19), studied the effects of acesulfame-k consumption by female rodents and found an association between acesulfame-k consumption and a reduction in pregnancy length, with adverse outcomes reported in offspring, including reduced fetal growth and hypoglycaemia. These findings mirror findings from observations studies, with associations between artificial sweetener use and pregnancy complications and increased childhood BMI. However, other studies have found contradictory conclusions, with reports of NCS consumption having no effect on fertility in human studies or animal models (20–22).

Although the link between maternal diet and offspring health has been well established, increasing evidence showing paternal diet at the conception period can program offspring health through direct pathways including changes to sperm and testicular epigenetic regulation, and indirectly through seminal plasma concentration (23). In addition, there is increasing evidence of paternal programming affecting female reproduction by altering placental gene expression and development (24, 25). Therefore, further research is needed to understand the etiology of diet and fertility, and to establish effective treatment and preventative strategies (26).

As consumption of NCS continues to rise and with male infertility contributing to half of infertility cases globally, we conducted a systematic review to critically examine the current evidence for an association between intake of non-caloric sweeteners and their impact on male reproductive parameters. Given the lack of information in a human setting we examined the role of NCS in rodent models.

## Materials and Methods

This review was carried out in accordance with the Preferred Reporting Items for Systematic Reviews and Meta-analysis (PRISMA 2009) guidelines (27). The protocol was developed in accordance with the Systematic Review Center for Laboratory animal Experimentation's (SYRCLE) guidelines. The population, intervention, comparator, outcome (PICO) question to be addressed in this study was framed as follows: what is the impact of NCS consumption vs. non-NCS consumption on male reproductive parameters, including sperm quantity (sperm count) and quality (motility, viability and morphology) in rodents? For this review, we included studies in mice and rats.

### Search Strategy and Study Selection

The following databases were searched: Pubmed, Web of Science, Embase and Scopus without time restriction. A gray literature search was also conducted in the following databases: Open Gray, Gray Literature Report, Zetoc, Proquest and Mednar. The Clinicaltrials.gov database and University College Dublin (UCD) Library were also searched for relevant studies. Searches started on 2nd February 2021 and concluded on March 1st, 2021. An extensive search strategy was constructed using keywords, related synonyms, and medical subject headings (MeSH). The final search strategy can be found in Supplementary Table S1. No date restrictions were applied. References were imported into Mendeley reference management software (v 1.19.4) and exported into Covidence systematic reviews production tool for screening and inclusion/exclusion.

Screening for inclusion was performed by two independent reviewers (MLK and FMA). Where conflicts arose a third reviewer made a final decision (CMR). Titles and abstracts were screened according to the predefined inclusion/exclusion criteria seen in Table 1 and Supplementary Table S1. Only intervention studies describing rodents (rat or mouse) consuming non-caloric sweeteners were included. No limitation was placed on NCS dosage. NCS had to be administered orally through the diet or by gavage to male rodents. Therefore, studies administering non-caloric sweeteners *via* injection were excluded. The NCS were defined as any artificially produced or natural sweetener with negligible energy content, therefore sugar alcohols were excluded. Studies were included if sperm quality, sperm concentration or pregnancy success were reported.

**Table 1:** Eligibility criteria.

*Inclusion criteria*
•Intervention studies •Male rodent models (rat or mice) •Exposure must be a non-caloric sweetener •Non-exposed control group •Sperm quality, sperm concentration, pregnancy success as primary outcomes
*Exclusion criteria*
•Non-intervention studies •Non-English studies •Nutritive sweetener used, to include sugar alcohols •Absence of a non-caloric sweetener control group •Non-caloric sweetener used in combination with additional dietary supplements or drugs •Type of sweetener used not specified •Sweeteners administered *via* injection •No relevant outcomes/secondary outcomes only reported

*Inclusion and exclusion criteria prioritized for title and abstract, and full text screening*.

### Data Extraction

The data was extracted by reviewer 1 (MLK). Data extracted from selected studies included bibliographical data such as first author and year and experimental data such as intervention (NCS type, dosage, route of administration and control used), animal characteristics (rodent species, strain, and age), sample size, study duration, main results and study limitations were extracted using Excel. For quality control, a random selection of the data (33% of studies) was checked for errors by reviewer 2 (FMA). If data in included studies was not reported, reviewer 1 (MLK) attempted to contact the corresponding author. Characteristics and findings of each study are illustrated in Table 2.

**Table 2:** Main characteristics and findings of the included studies.

**Author & Year**	**Sweetener used**	**Control group**	**Sweetener dosage & route of administration**	**Study population and age**	**Sample size**	**Study duration**	**Principal findings**	**Secondary outcomes**	**Limitations**
Anbara et al. (34)	Aspartame	0.5 ml saline	4 groups: C, LD, MD, HD: 40, 80 and 160 mg/kg. Oral Gavage	NMRI Mice Age: 8–10 weeks	36	90 days	Reduced sperm count in dose-dependent manner*, reduced sperm motility in MD and HD groups*, increased abnormal sperm morphology and DNA sperm damage in MD and HD groups*, effect in sperm viability in MD and HD groups, decreased sperm survival rate in MD and HD groups*, decreased Johnsen's score*	Increase in testicular capsule thickness in HD group*, decrease in seminiferous tubules diameter and germinal epithelium height in MD & HD groups*	Oral gavage administered study
Curry et al. (30)	Rebaudioside A	Standard chow & water	4 groups: 0, 1,506, 3,040, and 5,828 mg/kg/day weeks 0–12 and 0, 698, 1,473, and 3,147 mg/kg/day week 13. Diet *ad libitum*	Wistar Rats Age: 6 weeks	80	13 weeks	No effects on either spermatogenesis/testicular atrophy were detected on microscopic evaluation	Decreased epididymal weights in HD group*	Very high dosage - not translatable to human intakes, dose changed on final week of study. No data was provided on spermatogenesis findings^a^
Curry et al. (30)	Rebaudioside A	Standard chow & water	4 groups: 0, 586, 975, and 2,048 mg/kg/day. Diet *ad libitum*	Wistar Rats Age: 40–46 days	128	10 weeks	No effect on cauda epididymis/testis sperm count in HD group vs. control, small decrease in sperm motility in group 2 (586 mg/kg/day), no effect on sperm morphology between HD & control group	No effect on weights, macroscopic and microscopic examinations of epididymis and testis	Very high dosage - not translatable to human intakes, sperm count, and morphology only Reported for control & HD. Females in same group as males - gestational data not included
Gong et al. (37)	Saccharin	Standard chow & water or Sucrose	4 groups: Feed (%): C: 0, HD: 0.080, MD: 0.020, LD: 0.005. Water (mM): HD: 7.0, MD: 3.5, LD: 0.7. Diet *ad libitum*	ICR Mice Age: 8 weeks	84	35 days	Dose-response manner effect seen. In HD group: Reduced sperm count*, decreased *n* of rapid sperm and increased *n* of immotile sperm*, increased frequency of sperm tail and head abnormalities, lower sperm viability* Positive reproductive effect seen in MD and LD groups	HD: damage from the periphery to lumen of seminiferous tubules vs. control, wide-area seminiferous epithelial cells exfoliated from the basal layer of tubules, clusters of discohesive spermatogenic cells visible	
Kille et al. (32)	Sucralose	Distilled water	4 groups: Control, 6-chloroglucose 24 mg/kg/day, Sucralose 500mg/kg/day, TCDS, 100 mg/kg/day, oral gavage	Sprague-Dawley Rats Age: Only stated as “adults”	40	28 days	No significant inter-group differences in mean sperm concentration between groups including NCS and C	N/A	Very high dosage - not translatable to human intakes. Oral gavage route administered. Sperm motility, morphology or viability not assessed
Machemer et al. (35)	Saccharin	0.5 ml of demineralized water/ 20g/ weight	2 groups: Control, 5g/kg/day Oral solution in water	NMRI/BOM Mice Age: Only stated as “males weighed 25–30 g”	20	5 days	No significant differences in fertilization rate, pre or post-implantation rate of NCS group vs. control	N/A	Short study duration, it was acknowledged that there are known difficulties involved in the evaluation of pre-implantative loss
Melis et al. (31)	Rebaudioside A	saline	2 groups: Control, 66.7g of dried leaves/100 ml final solution, Gastric Tubing	Wistar Rats Age: 25–30 days	20	60 days	Reduced sperm concentration in NCS group*	Reduced cauda epididymis, seminal vesicle + testis weight in NCS group*	Sperm motility, morphology or viability not assessed, gastric tubing administered route
Otabe et al. (33)	Advantame	Standard chow and water	2,000, 10,000, or 50,000 ppm/ 164, 833, and 4,410 mg/kg bw/day, diet *ad libitum*	Crl:CD (SD) IGS BR Rats Age: 5 weeks	136	5 weeks	No significant effects in sperm count, motility or abnormal sperm morphology between HD and control	N/A	Large NCS dose - not translatable to humans. Fertility analysis was carried out on control and HD only
Rahimipour et al. (36)	Saccharin	Standard chow and water	2 groups: control, 0.2% w/v, water *ad libitum*	BALB/CMice Age: 10 weeks	14	35 days	Reduced sperm count, sperm motility and sperm viability in NCS group*, Increased abnormal sperm morphology apoptosis and rates of sperm with DNA fragmentation in NCS group*	N/A	Small sample size

*C, control; HD, High dose; MD, Medium dose; LD, Low dose.*

^*^Significance at the P <0.05 level.

*^a^Data not provided*.

### Study Quality Assessment

#### Risk of Bias

The included studies were assessed for internal validity using SYRCLE's Risk of Bias (RoB) tool, adapted from the Cochrane RoB tool, and has been developed for use in systematic reviews on animal models to avoid inconsistencies when assessing quality of animal intervention studies, as they differ from randomized control trials (RCT) (28). The tool contains 10 items to investigate sources of bias such as selection bias, performance bias, detection bias, attrition bias, reporting bias and other biases. These items were assessed as “low risk of bias,” “high risk of bias” or “unclear” using the signaling questions provided (28). Quality assessment of these studies are shown in **Figure 2**.

## Results

### Study Selection

**Figure 1:**
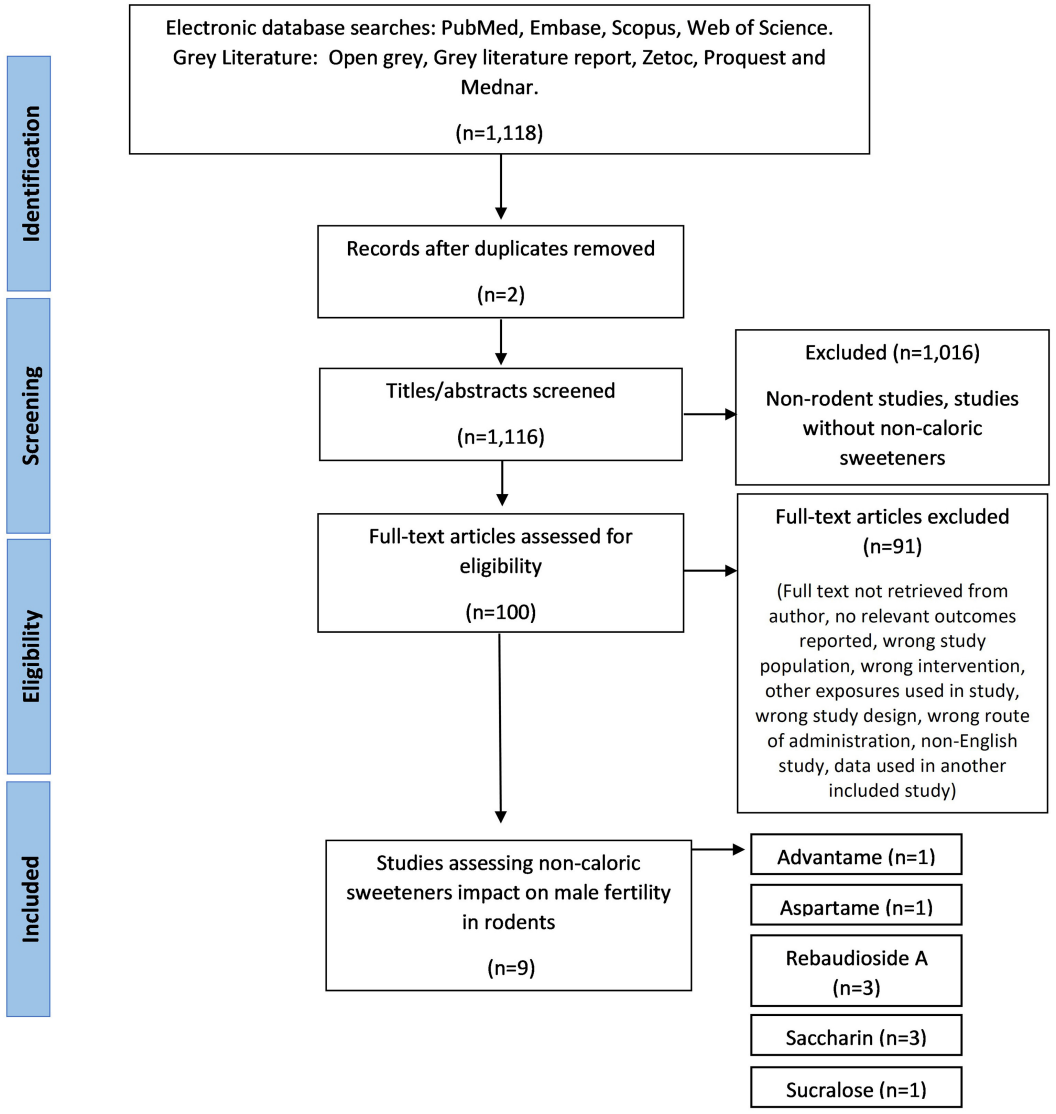
The PRISMA flow diagram for the systematic review detailing the database searches, the number of abstarcts screened, and the full texts retrieved.

Initial searches of databases produced 1,118 papers, of which 1,116 remained after duplicates removed. Phase one of screening, based on title and abstract, excluded 1,016 papers. Phase two of screening was based on full text review and resulted in the exclusion of 91 papers, which resulted in nine included papers (Figure 1). During full-text screening, 38 papers were excluded as the full text was not retrieved by the author, 13 papers were excluded due to the NCS exposure in the wrong population (such as female rodents), 11 papers were excluded for using the wrong intervention (including sugar alcohols and nutritive sweeteners), eight papers were excluded due to the wrong study design (e.g., non-intervention studies), eight papers were excluded due to irrelevant outcomes reported, six papers were excluded due to other exposures used along with the NCS (e.g., caffeine), four papers were excluded for being a non-English study, two papers used the wrong route of administration and one paper was excluded as the data was already present in an included study.

### Study Characteristics

Study characteristics varied for rodent species and strains, age, weight, type, dosage, and timing of NCS administered. In relation to species and strain, Wistar rats were used for most studies (29–31), followed by two studies in Sprague-Dawley rats (32, 33). In mice, NMRI strain were the most used (34, 35) one study used ICR mice, and one study used BALB/C mice (36). Five types of sweeteners were used (rebaudioside A—three papers, saccharin—three papers, advantame—one paper, aspartame—one paper, and sucralose—one paper) and were mostly administered to the rodent's feed or drinking water (29, 30, 33, 35–37) (see Table 2). Two studies used oral/aqueous gavage (32, 34) and one study used gastric tubing to administer the NCS (31).

Considerable variation in NCS dosage was used, ranging from near the acceptable daily intakes for humans (ADI) (34), to excessive intakes equivalent to over 100 times greater than the ADI for humans (33). Timing and duration of the intervention also varied widely in the studies, ranging from 5 days to 90 days (Table 2).

### Male Fertility Outcomes

Results of the finalized search strategy are presented in Table 2. Due to the heterogeneity between the included studies, it was not possible to conduct a meta-analysis. Sperm concentration was recorded in the majority of studies (30–34, 36, 37) and sperm quality was assessed in over half (5/9) of the studies (30, 33, 34, 36, 37). Pregnancy success was only studied in one paper (35). Reproductive organ weight or damage was recorded in 5 studies (29–31, 34, 37). One study reported outcomes without providing data (as indicated in Table 2. with superscript a) (29).

### Sperm Concentration

Seven papers reported on sperm concentration as seen in Table 2. Methods of assessing sperm concentration were varied, resulting in a differing of sperm count outcomes, as shown in Table 3, with studies reported sperm counts as millions/ml or g. Four papers recorded a significant decrease in sperm count in rodents fed NCS, compared to control fed rodents (31, 34, 36, 37). Two studies reported reduced sperm count in a dose-dependent manner, with the most significant decrease in sperm count seen in high or middle-dose groups compared to low-dose and control groups (34, 37). One study containing aspartame (34), two studies containing saccharin (36, 37) and one study containing rebaudioside A (31), concluded that consumption of NCS decreased sperm count compared with controls. One study reported no effect of NCS consumption on spermatogenesis, however no data was provided on sperm count in control or treatment groups (29). Three studies with negative effects of sperm concentration used mice (34, 36, 37), two studies administered the NCS in diet *ad libitum* (36, 37), one by oral gavage (34) and one by gastric tubing (31). Three papers containing; rebaudioside A (30), sucralose (32) and advantame (33), reported no significant effects of NCS on sperm concentration compared with controls, upon completion of the intervention duration. All studies with no significant decrease in sperm count used rats as the animal species. Two studies administered NCS in diet *ad libitum* (30, 33) and one by oral gavage (32), with the highest doses administered to treatment groups ranging from 500 (32) to 44,100 mg/kg/bw (33).

**Table 3:** Data of included studies reporting sperm count outcomes in NCS studies.

**Author**	**NCS used**	**Measurement used**	**Method used**	**Data presented**	**Sperm count control group**	**Sperm count NCS group**		
						**LD**	**MD**	**HD**
Anbara et al. (34)	Aspartame	×10^6^/ml	Standard hemocytom-eter method	Table. mean ± SD	34.7 ± 1.7*	31.6 ± 1.4*	27.4 ± 1.8*	19.2 ± 1.5*
Curry et al. (30)	Rebaudioside A	Millions/g for c.a and t	NR	Table. mean ± SD	790 ± 182^ce^ 217 ± 34^t^	**LD**	**MD**	**HD**
						NR	NR	790 ± 171^ce^ 213 ± 31^t^
Gong et al. (37)	Saccharin	×10^6^/ml	Neubauer chamber	Bar chart. approx (~) mean	~70*	**LD**	**MD**	**HD**
						~70*	~75*	~55*
Kille et al. (32)	Sucralose	×10^8^/ml	Standard hemocytom-eter method	Table. mean ± SD	0.8 ± 0.2	0.8 ± 0.2
Melis et al. (31)	Rebaudioside A	no./ml ×10	NR	Table. mean ± SEM	173.7 ± 8.0	117.7 ± 9.0*
Otabe et al. (33)	Advantame	Millions/g for c.a and t	Hamilton Thorne IVOS Computer Assisted Sperm Analyzer (CASA) v 12.0.	Table. mean ± SD	587 ± 127^ce^ 138 ± 32^t^	**LD**	**MD**	**HD**
						NR	NR	553 ± 162^ce^ 127 ± 34^t^
Rahimipour et al. (36)	Saccharin	×10^6^/ml	Makler chamber	Table. mean ± SD	17.7 ± 1.1	12.8 ± 2.8

*C, control; LD, low dose; MD, middle dose; HD, high dose.*

^*^Significant difference between groups (P <0.05).

*c.a, cauda epididymis; t, testis; NR, not recorded*.

### Sperm Quality

Over half of the studies (5/9) reported on sperm quality as seen in Table 2 (30, 33, 34, 36, 37), including sperm motility, viability, morphology and sperm DNA integrity and were mostly expressed as percentages (%). Three papers reported a significant decrease in sperm quality upon completion of a NCS intervention (34, 36, 37). Two studies reported a decrease in the percentage of motile sperm, normal sperm morphology and sperm viability in NCS groups compared with controls (34, 37). One paper investigating saccharin, quantified different motility variables, including rapid, slow, non-progressive and immotile sperm and found the number of rapid sperm in the high dose-saccharin group [feed %: 0.08, water (mM): 7.0] was significantly decreased, while the number of immotile sperm was significantly increased (37). One study investigating aspartame, reported a 23% decrease in sperm motility and a 17% decrease in sperm viability compared to low dose or control groups (34). Sperm morphology was reported in five studies (30, 33, 34, 36, 37), of which, three studies saw negative effects, including increased abnormal sperms (34, 36), increased sperm with DNA damage (34) and increased sperm head and tail abnormalities (37) following the consumption of NCS. Two studies containing saccharin (37) and aspartame (34), reported effects in sperm quality in a dose-dependent manner, with high or middle dose NCS groups affecting normal sperm morphology and viability, compared with lower NCS dosage groups and control. There was a 14% increase in sperm DNA damage in the high aspartame dose group compared to controls (34), while in the saccharin intervention study, there was an increased frequency of sperm tail abnormalities in the high-dose saccharin group compared to all lower saccharin or control groups (37). In another study investigating saccharin, consumption of the NCS increased sperm DNA fragmentation and apoptosis, compared to the control group (36). Only one study reported seeing a positive reproductive effect of NCS consumption in mice (37). This study showed that while the high-dose saccharin group (feed %:0.080, water: 7 mM) induced a negative reproductive effect on rodents, the low (feed %:0.005, water: 0.7 mM) and middle (feed %: 0.020, water: 3.5 mM) dose saccharin groups induced a positive reproductive effect, with no adverse effects on sperm quality (37). All studies using mice reported negative effects on sperm quality, compared to two studies using rats, who observed no negative effects on sperm quality.

### Pregnancy Success

Only one study investigated pregnancy success as shown in Table 2, following saccharin consumption in males (35). Mice were given saccharin for 5 days and then mated with untreated females to assess fertility and implantation loss. The study found NCS did not impair fertility or mating capacity, compared with controls. Pre and post-implantation loss was within the normal range of the strain in NCS treated mice.

### Reproductive Organ Changes

Five of the nine studies reported on reproductive organ effects following consumption of NCS (29–31, 34, 37), as shown in Table 2. Two studies investigating rebaudioside A reported reduced cauda epididymitis weight in NCS groups compared to controls (29–31). Two studies investigating aspartame (34) and rebaudioside A (31), reported a reduction in seminal vesicles and seminiferous tubule diameter of NCS treated rodents, compared with controls, with one further study reporting damage to seminiferous tubules also in saccharin high-dose groups (37). Two studies reported changes to the testes in NCS groups, one study reported increased testicular capsule thickness in the aspartame treated high-dose group (34) and reduced testis weight in rats following consumption of rebaudioside A (31). Other reproductive organ changes following NCS consumption included decreased germinal epithelium height in middle and high dose aspartame groups (34) and wide area exfoliation of seminiferous epithelial cells from the basal layer and visible clusters of discohesive spermatogenic cells in a saccharin study (37). Two studies investigating rebaudioside A, reported no effect on reproductive organ weight or changes (30) or testicular atrophy upon microscopic evaluation upon the study completion (29). Four of the nine studies did not report on reproductive organ changes (32, 33, 35, 36).

### Study Quality Assessment

#### Risk of Bias

**Figure 2:**
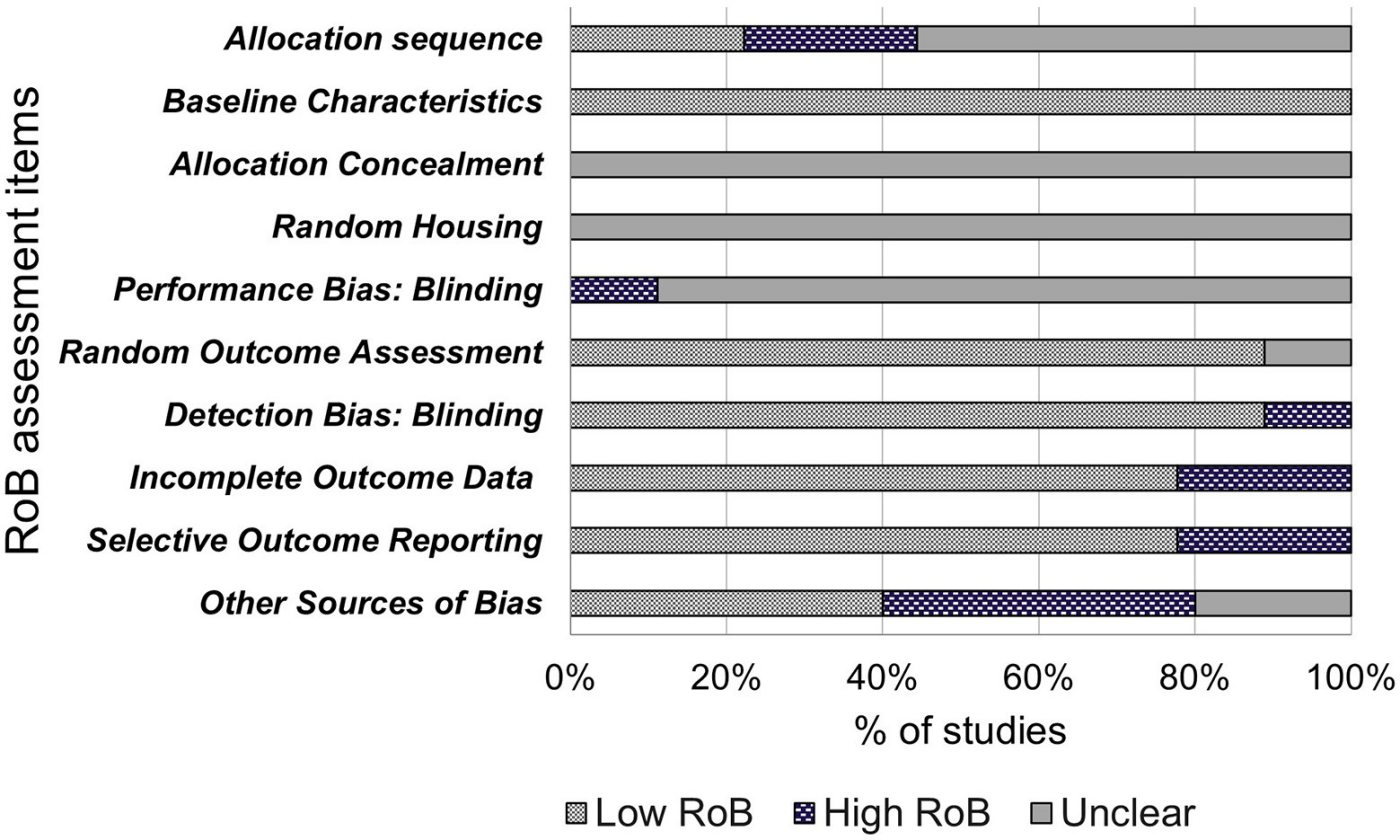
Risk of bias.

The results of bias assessment are presented in Figure 2. Results are reported as the percentage of papers per item categorized as low risk of bias (yes), high risk of bias (no) or unclear risk of bias. Items 1–3 related to selection bias. Only two (22%) of the included papers reported randomization of animal allocation using methods such as random number tables or labeling for randomization. Five papers reported using randomization yet failed to describe the method of randomization and were therefore judged as “unclear.” The remaining two papers did not report randomization. All nine papers (100%) described similar baseline characteristics for age and weight. None of the nine included papers reported concealing allocation to NCS groups from the researchers or investigators. Random bias (item 4) describes the measures used to house the animal randomly within the animal room or blinding of investigators from knowing the intervention received by each animal (28). This bias was judged as unclear as none of the included papers described randomly housing animals within the animal room. One paper was judged as “high” for performance bias (item 5), as each animal was identifiable to the investigators during the experiment with a tag. Items 6 and 7 relate to detection bias and describes the measures used to animals selected for outcome assessment and the outcome assessor themselves being blinded from intervention animals (28). Eight of the included studies were judged as “low” for outcome assessment as all animals were used in the outcome assessment and 1 was judged as “unclear” as it was not clear the quantity of included animals for outcome assessment. Eight papers were judged as “low” for detection bias as the reviewers judged that the outcome is not likely to be affected by blinding due to outcome assessment methods being the same in both the control and treatment groups. One paper was judged as “high” as investigators only examined organ morphology in high dose groups if rodents showed signs of reduced fertility. Additionally, sperm morphology was not assessed in lower dose groups. Item 8 refers to attrition bias. Seven papers were judged as “low” bias for completeness as any missing animals were accounted for and all main outcomes were assessed. Two papers were judged as “high” bias due to not giving indication of reason for excluding data for low and middle-dose NCS groups. Due to this exclusion, two papers were also judged as “high bias” for selective outcome reporting (item 9) due to fertility analysis data only reported for control and high dose-NCS groups, with low and middle-dose groups data not reported. Seven papers were judged as “low” for including all outcome data. Four (44%) papers were assessed as potential risk of bias due to industry funding sources (item 10), two were unclear and four were “low” bias due to no other bias detected by reviewers.

## Discussion

The aim of the current paper was to undertake a systematic review of animal studies to evaluate the effect of non-caloric sweeteners on fertility in male rodents. Included were papers of NCS administered to male rodents, with sperm quality, concentration and pregnancy success assessed as outcomes. Results of fertility outcomes, due to NCS consumption was varied. The majority of studies (56%) (29, 30, 32, 33, 35) reported no observed effect on male fertility following the consumption of NCS compared to controls. The variability in results could be explained by the disparity in study duration, sample size, rodent strain, and assessment methods. NCS dosage was wide-ranging among the studies, with a large proportion of studies reporting excessive doses, which could also account for the variability of results. For homogeneity of results, future researchers should use concentrations of NCS appropriate to the typical concentrations consumed by humans.

Nevertheless, four studies (44%) (31, 34, 36, 37) investigating the NCS; aspartame, saccharin and rebaudioside A, did report significant effects of NCS consumption on reproductive parameters, with all four studies reporting a significant reduction in sperm count in NCS groups compared to controls. However, the marked differences in sperm concentration analysis and reference values reported must be noted. Previous studies have shown significant differences in sperm sample count using separate methods, with both the Makler and Hemacytometer counting chambers shown to overestimate sperm concentration (38, 39). The lack of standardized protocols around sperm count analysis has made it difficult for this review to establish the impact of NCS on sperm concentration, while also highlighting the need for a standardized protocol and quality control. The WHO has published semen analysis protocol manuals as recently as 2021 and is recognized as the gold standard for human semen analysis worldwide, intended to maintain and sustain the quality of analysis and the comparability of results (40). Only two of the seven included studies that reported on sperm concentration in this review referenced using the WHO Hemacytometer sperm count method (34, 37). It is therefore difficult for this review to determine the effect of NCS on sperm concentration. Moreover, there has been a lack of clarity surrounding normal reference values for sperm count. The reference values for sperm concentration have been cited by the WHO as 15–259 × 10^6^/ml and described in a report by Cooper et al. (41). Issues on reference values for sperm count have been further highlighted in a systematic review, carried out by Patel et al. (42), which found that sperm concentration shows the greatest intra-individual variation, and sperm viability and total motility showing the least variation. Therefore, it may be more appropriate for studies to focus on sperm viability and motility as a more accurate measure of fertility.

Two studies Anbara et al. (34) and Gong et al. (37), observed negative reproductive effects in a dose-response manner, suggesting that, as NCS dose rises, reproductive parameters decrease, resulting in reduced sperm count, motility and increased abnormal sperm morphology, including increased sperm head and tail abnormalities, decreased overall normal sperm morphology and increased sperm DNA damage. Mechanisms for reduced fertility in the identified studies were predominantly undetermined. However, one study identified reduced fertility parameters were induced by increased production of free radicals, induction of oxidative stress and weakening of the antioxidant defense system, following the consumption of high-dose aspartame (34). Previous studies in rodents have also established a link between NCS consumption and oxidative stress (43, 44), which is consistent with the findings in this study. Spermatozoa are particularly vulnerable to the deleterious effects of reactive oxygen species (ROS) due to large quantities of unsaturated fatty acids located in their cell membranes, which results in lipid peroxidation, increased permeability due to loss of membrane integrity, reduced sperm motility, DNA damage and apoptosis (45). The study also reported aspartame increased serum malondialdehyde (MDA) levels and Nitric oxide (NO), indicators of phospholipid breakdown and markers of oxidative stress, as well as increased serum total antioxidant capacity (TAC) and blood catalase activity (CAT), constituents of antioxidant defense (34). Another included study (36) failed to reach any definitive conclusions on the effect of saccharin on fertility, however acknowledged previous research that shows saccharin enhances ROS production and hypothesized that the saccharin-treated mice underwent oxidative stress, resulting in sperm DNA damage, affecting sperm parameters (46).

One study concluded the effects in fertility were potentially due to the suppression of Taste receptor T1R3 and G protein alpha-gustducin (Galpha), observed in the high dose saccharin rodent group (37). Research has shown that T1R3 and gustducin α-subunit GNAT3 knockout mice, leads to male-specific sterility, giving rise to malformed and immotile sperm, highlighting the potential significance of these taste receptors as chemosensors which play an essential role in maintaining reproductive parameters (47). Previous *in-vivo* and *in-vitro* studies have also shown that NCS bind to intestinal taste receptors, disrupting intestinal tight junctions and result in reduced barrier function and leakage across the intestinal epithelium (48, 49). However, less is known about the physiological role of extraoral sweet taste receptors, particularly in the testes (50). Furthermore, the extent of NCS bioavailability in reproductive tissue requires further study. It is possible that each NCS may elicit different physiological effects due to their contrasting absorption, metabolic and excretory pathways following ingestion. For example, saccharin and acesulfame K enter systemic circulation following absorption and are distributed to body organs *via* the blood, until they are excreted in urine or feces. Aspartame is absorbed and metabolized by the liver before breaking down further into methanol, aspartic acid, and phenylalanine. Stevia is metabolized by the liver and excreted in the urine, while sucralose is poorly absorbed, undergoes little metabolism, and is excreted primarily unchanged in the feces (51). Due to these differing metabolic pathways, it is plausible that the bioavailability in certain body tissues, including the testes is also variable according each NCS. In addition, even if certain NCS reach the reproductive tissues, the mechanisms involved in inducing any positive or negative reproductive effects, remains poorly understood. However, some studies have shown that NCS reach these tissues following consumption. Gong et al. (37) has previously shown an increase in the sweet-sensing molecules T1R3 and its subunit Gx, following saccharin consumption in mice, while others have shown traces of saccharin radioactivity remain in many tissues after 72 h, including the testes, indicating that saccharin may have specific biological functions in the male reproductive tissues (52). Future studies are needed to fully understand the bioavailability of NCS in tissues following absorption and their impact on reproductive tissues, including sweet taste receptor expression.

One included study investigated rebuadioside A, yet the study failed to establish a mechanism for significant decreases in sperm concentration observed in the NCS group (31). Hypotheses included impaired production of sperm by the testis, the effect of NCS on the epididymal mechanism of fluid absorption by vasodilation, or by decrease in plasma testosterone. A previous study carried out by the author showed oral administration of an aqueous extract of Stevia to rats induced systemic and renal vasodilation (53), which may explain the effect on fertility. Previous research has shown vasodilators induce spermatotoxic or antispermatogenic effects in male rats (54). There have been reports that Paraguayan Indians consumed tea brewed from the stevia plant as an oral contraceptive (55), however subsequent studies have yielded contradictory results, which is also reflected in the variation in the results of the included studies on rebuadioside A (29–31). A decrease in sperm concentration was reported in one study (31), while one study observed a non-significant small decrease in sperm motility in the lower dose NCS group (30). A reduction in reproductive organ weight was reported in two of the three studies (29, 31). Three of the included studies (31, 34, 37) also explored the influence of NCS on androgenic effects in the rodents and assessed serum testosterone as a potential mechanism of reduced sperm parameters. Testosterone is synthesized from Leydig cells and is essential for spermatogenesis maintenance (56). Previous research observed high-dose NCS consumption significantly reduced the number of Leydig cells, which may negatively impact their ability to synthesize testosterone (56), while other research has also shown NCS consumption reduces testosterone levels (57, 58). Future studies investigating diet and male reproductive outcomes should include serum testosterone and/or leydig cell analysis, in addition with sperm parameter assessment, as a potential mechanism in decreased male fertility.

Pregnancy success outcomes were difficult to assess in this review, due to the lack of studies included. The one included study on fertilization rate (35) saw no effect of saccharin on male mice fertility, however this study acknowledged difficulties in evaluating pre-implantation loss and was conducted over a short time period of 5 days. A short-term study such as this, may present difficulties in interpreting fertility outcomes, as it takes four spermatogenic cycles, the equivalent to 35 days, for spermatogonia to develop into spermatozoa in mice (59). Furthermore, Kille et al. (32), conducted their study over a 28 day period in Sprague-Dawley rats, which also fails to cover the rat spermatogenic cycle of 56–58 days. Moreover, when assessing sperm parameters in rodents, it is important to use sexually mature animals for optimal results. The use of immature rodents can present some challenges in analyzing effects on sperm parameters, as shown in a previous study by Saksena et al. (60), where no sperm was observed in the male reproductive tract until 42 days of age, and the peak reproductive period was found to be between 100 and 270 days of age. In this review, the majority of included studies assessed spermatozoa after rodents reached sexual maturity, however one study assessed rat spermatozoa just as they reached sexual maturity of 10 weeks (33). Other studies have also identified that the age of rats is key to maximal sperm reserves, with advanced age causing decreased motility in advanced age rats (61, 62). Therefore, male fertility studies should consider covering the full spermatogenic cycle of each animal model used, as well as rodents during their peak reproductive period as part of their experimental design to yield robust and conclusive fertility results.

In addition, 56% of included studies assessed reproductive organs for morphological differences, of which, 44% reported reproductive organ damage or change in measurement, following NCS consumption. Certain morphological analysis however, such as weighing the testis, provides limited information in comparison to spermatogenesis analysis (63). Testicular weight in rodents can also vary with age, strain, housing conditions and diet, for example in CD-1 mice, testicular weight was shown to decrease with age (64), whereas in Sprague-Dawley rats, testicular weight increased with age (65). Furthermore, the WHO does not list histological analysis of reproductive organs as a measure of fertility and the authors of this review did not include it in our primary outcomes (66).

This review demonstrates that due to the heterogeneity in study design, NCS dosage, species used and variation in the analyses of sperm samples in the included studies, it is difficult to determine if NCS consumption causes any negative effects on male reproductive parameters. The distinct role and mechanisms of NCS in male fertility remains unclear. Out of the four studies noting an effect of NCS on male fertility, only one study provided mechanistic insights into the observed decline in reproductive parameters. It is apparent that future research should place emphasis on investigating the precise mechanisms contributing to any potential negative reproductive outcomes observed and if consumption of NCS at the human ADI can induce oxidative stress or affect taste receptor expression in the testes. The emerging research in taste receptors in sperm biology, particularly their key functional role in the process of fertilization, may help determine these mechanisms, given that these NCS bind to sweet taste receptors and should also be considered in future research. In addition, only five included studies assessed sperm motility and morphology in addition to sperm concentration and only one study investigated pregnancy success. The WHO manual for examining and processing human semen, with emphasis on assessing sperm motility, vitality and morphology, in addition to sperm concentration, due to their importance in sperm function, should be used as a gold standard in sperm analysis (67). Assessing multiple reproductive parameters, including fertilization success with untreated females in future studies may help obtain more conclusive results on NCS and their contribution to male fertility, as well as report findings more relevant to human reproductive health. Of the included studies, saccharin and rebaudioside A dominated the studies, yet acesulfame-K, which is the most consumed NCS in the Irish food supply (61) and in Europe (68) was not included. In addition, only one study on sucralose was included, which is the most widely consumed NCS in North America (69). The current analysis was also challenged by the variability in NCS type and dosage, study duration and animal strain, which is therefore difficult to apply to human health. Moreover, there was significant risk of internal bias across most studies, mainly due to a lack of reporting of allocation concealment, randomization and blinding.

Due to the rising consumption of NCS as a sugar substitute and the growing concern of infertility, affecting ~15% of couples worldwide (26), the impact of NCS on male fertility was considered a relevant outcome for this review. With only one study conclusively finding oxidative stress to be a physiological driver of male infertility, and only five studies carrying out sperm analysis to include motility and morphology, and one assessing fertilization success, there is a clear gap in the research for future studies to explore.

Within the current review, the limitations of rodents as models for human NCS research must be acknowledged. Rodent preferences for sweeteners are dissimilar to humans, for example, both rats and mice dislike the taste of aspartame and sucralose, while rats perceive saccharin as bitter rather than sweet (70). Consideration should be given in the studies where rodents had free access to NCS, as seen in 6 of the included studies (29, 30, 33, 35–37), Future studies should carefully monitor and measure the intakes of food or water in which the NCS are added and offered to rodents *ad libitum*, and record any inconsistencies in intakes between the treatment and control groups., A high risk of bias was demonstrated for the 44% of the studies due to funding sources. There is potential bias in the NCS industry, with industry-sponsored reviews more likely to achieve favorable results compared to non-industry reviews (71). In this review, we found all (100%) of the industry-funded interventions reported no effects of NCS consumption of male fertility. Future animal studies should ensure appropriate randomization and blinding, while adhering to guidelines of reporting animal studies, to improve quality of studies, such as the ARRIVE guidelines, which are considered the gold standard for improvement of transparency and reproducibility in animal studies (72). A final limitation of this review was the high number of inaccessible manuscripts for inclusion, the majority of which consisted of older studies and published between 1970 and 1990.

This review has several strengths, including a comprehensive study quality assessment, using the SYRCLE's risk of bias tool, a favorable instrument to establish consistency and avoid discrepancies in measuring risk of bias in animal intervention studies. This study also had a robust and thorough search strategy, including a comprehensive gray literature search, and had transparency in methodologies and used the Covidence tool for conducting systematic reviews, enabling accurate reporting. To our knowledge, this is the first fully comprehensive systematic review summarizing all available evidence on the effect of NCS consumption on male fertility in a rodent model.

## Conclusions

It is still largely undetermined if NCS have the potential to affect male fertility, with some studies showing significant effects on reduced sperm quality and concentration in rodents consuming aspartame, saccharin and rebaudioside A. There may be a dose-dependent manner of NCS effects, with higher intakes associated with worse fertility outcomes, compared with lower NCS intakes and control groups. The specific effects of NCS on fertility have been conflicting and the available studies have been heterogeneous in terms of the study duration, assessment methods and outcomes, sample size and doses evaluated. Although oxidative stress has been identified as a likely driver of infertility in one study, however there may be other mechanisms at play, including taste receptor expression in the testis and androgenic effects. Further research is needed to assess the role of NCS in male infertility both in animal models and human studies. Future studies should use the WHO protocol, considered the gold standard for semen analysis and also ensure accurate reporting of randomization and blinding, as well as adherence to the ARRIVE guidelines for improvement of transparency and reproducibility in animal studies (67). Ensuring animal fertility research covers the full spermatogenic cycle appropriate to the rodent species can also identify the full potential effect of NCS on fertility and mitigate acute effects. Finally, NCS concentrations at the human ADI should also be considered for translational relevance for human health.

## Data Availability Statement

The original contributions presented in the study are included in the article/Supplementary Material, further inquiries can be directed to the corresponding author.

## Author Contributions

Conceptualization and writing—review and editing: CR and MK. Methodology and data curation: MK, FM, and CR. Writing—original draft preparation: MK. All authors have read and agreed to the published version of the manuscript.

## Funding

This paper was funded by the UCD Wellcome Institutional Strategic Support Fund, which was financed by University College Dublin and the SFI-HRB-Wellcome Biomedical Research Partnership.

## Conflict of Interest

The authors declare that the research was conducted in the absence of any commercial or financial relationships that could be construed as a potential conflict of interest.

## Publisher's Note

All claims expressed in this article are solely those of the authors and do not necessarily represent those of their affiliated organizations, or those of the publisher, the editors and the reviewers. Any product that may be evaluated in this article, or claim that may be made by its manufacturer, is not guaranteed or endorsed by the publisher.
