# Supplementary material for: The Impact of Non-caloric Sweeteners on Male Fertility: A Systematic Review and Narrative Synthesis in Rodent Models
Source: Front Nutr. 2022 Jun 28;9:854074. doi: 10.3389/fnut.2022.854074 (PMC9274198; doi:10.3389/fnut.2022.854074)
Supplement: Supplementary file 1 [file Data_Sheet_1.docx]

Search Strategy

**1.1 Review Question/title**What is the impact of non-caloric sweeteners on male fertility?

**1.2 Aim of the research**To determine if non-caloric sweetener consumption has a negative impact on male fertility outcomes; pregnancy success, sperm quality, sperm quantity, pregnancy success

**1.3 Identifying key concepts: PICO**



**1.3.1 Search terms:**

| **Non-caloric sweeteners** | **Infertility** | **Male** | **Rodent** |
| --- | --- | --- | --- |
| - Non-caloric sweeteners - Sweetening agents - Non caloric sweeteners - non-nutritive sweeteners - Sweetener - High Intensity sweetener - Intense sweeteners - Sugar substitute - Acesulfame potassium - Acesulfame K - Ace K - Stevia - Stevioside - Stevia rebaudiana - Steviol glycoside - Rebaudioside A - Aspartic acid - Aspartame - Cyclamates - Advantame - Saccharin - Sucralose - Neotame - Sugar alcohols | - Infertility - Infertility, male - Infertile - Subfertile - Sterility - Contraception - Sperm - Spermatogenesis - Semen - Sperm parameters - Semen parameters - Sexual dysfunction, physiological - Reproduction - Preconception - Pre-pregnancy - Testicular - Testes - Testosterone - Sexual behaviour, animal - Pregnancy, animal - Fertilisation | - Male - Father - Paternal - Paternal exposure | - Animal(s) - Animal experimentation - Models, animal - rat - rats - mice - mouse - murine - muridae - murids |

There will be use of Boolean operators to narrow/broaden the search (AND/OR)

**1.4 Database Searches**

We will perform an electronic search of the following databases: PubMed, Embase, Scopus and Web of Science without time restriction. Manual searches of the reference list and citation of included articles and relevant reviews will also be performed to identify additional studies. Limits will be applied to english language and rodent models.

**1.4.1. Additonal Searches**

We will also carry out grey literature searches in the following databases: open grey, Grey literature report, Zetoc, Proquest and Mednar. The following conference proceedings will also be searched: Proceedings of the nutrition society, International conference of animal reproduction and fertility, Dohad world congress, European nutrition conference (FENS), ASN. Other searches will include Theses & Dissertations, Research Reports, Technical Reports.

**1.5 Collating & Extraction of studies**

Using a reference management software e.g. Mendeley. A group created will share references downloaded by two reviewers. These will then be extracted to Covidence for screening and voted for inclusion/exclusion. Title abstract dual screening will ensure thorough review of all studies. Bulk upload of PDFs for full text screening, selecting reasons for exclusion.

**1.6 Inclusion and Exclusion Criteria**

**1.6.1. Study Designs to be included:
*Inclusion criteria***Interventional Studies

***Exclusion criteria***
Non-interventional studies

**1.6.2 Animals/population
*Inclusion criteria***Rodent models (mice and rats)

***Exclusion criteria***Non-rodent models, human models, in vitro, ex-vivo, in silicio studies

**1.6.3 Intervention(s), Exposure(s)
*Inclusion criteria***
Exposure must be a non-caloric sweetener e.g., aspartame, ace-k etc.

***Exclusion criteria***Nutritive sweetener e.g. sucrose, fructose, female rodents fed exposure in addition to males prior to mating, absence of a non-caloric sweetener control group, non-caloric sweetener used in combination with additional dietary supplements or drugs, type of sweetener used not specified (unclear if sweetener is non-caloric or nutritive), sweeteners administered via injection, sugar alcohols.

**1.6.4 Control/comparison
*Inclusion criteria***Non-exposed control group: e.g standard chow & water or placebo, caloric control e.g. nutritive sweetener or sucrose, or same dietary intervention without Non-caloric sweetener

***Exclusion criteria***Exposed control group, absence of control males for comparison

**1.6.5 Outcome(s)
*Inclusion criteria***Primary outcomes: Sperm quality, sperm quantity, pregnancy success,

Secondary outcomes: weight of reproductive organs, litter size, gestation length, sexual and consummatory behaviour (mating, urge to mate etc)

***Exclusion criteria***No relevant outcomes reported (those not stated in inclusion criteria)

**1.7 Study selection**

***Procedure for study selection***

- **Step 1) Title and abstract screening:** 2 reviewers (MLK, FM) will independently screen the title & abstracts of identified articles for obvious exclusions. Any disagreements will be discussed between reviewers and if necessary, with a third party (CMR)
- **Step 2) Full text screening:** of the eligible studies, full text articles will be sourced, and the reviewers will independently review these articles in relation to the inclusion & exclusion criteria. Any disagreements will be discussed between reviewers and if necessary, with a third reviewer (CMR)

***Prioritise the exclusion criteria***

**Step 1) Title and abstract screening:**

1. non-rodent studies
2. Studies without non-caloric sweeteners

**Step 2) Full text screening**

- No appropriate control group comparison e.g. nutritive sweetener/no non-caloric sweetener group
- Female rodents in group also given non-caloric sweetener
- Additional drugs used in combination with sweetener
- Non-specified sweetener used (unclear if non-caloric or nutritive)
- No relevant outcomes reported
- Non-English Study

**1.8 Methods for data extraction**

Data will be extracted directly from the text, data tables and graphs. If data is not present in the article, the authors will be contacted. Onesearch will be searched if reviewers can’t get full text articles. One reviewer will extract data. For quality control, a random selection of the data will be checked for errors by the second reviewer. If discrepancies occur, they will be corrected based on the original text.

**1.8.1 Data to be extracted**

**Study design:** Study design, number of animals per group, control group and study duration.

**Animal model:** Rodent species and strain

**Intervention of interest:**
1. Type of dietary intervention: type and dosage of Non-caloric sweetener

2. Duration of NCS exposure

3. Mode/route of sweetener exposure (e.g. within food or beverage, type of chow, free access or timed exposure)

**Primary outcomes**: Pregnancy success, sperm quality, sperm quantity

**Secondary outcomes:** Reproductive organ weight, litter size, gestation length

**1.9 Risk of bias and/or quality assessment**

By use of syrcle ROB completed by the two reviewers. If any discrepancies arise, they will be resolved through discussion, and a third reviewer (CMR) if needed.
